# Cincinnati Obstetrical Society

**Published:** 1895-08

**Authors:** 


### *SOCIETY REPORTS.*

---

### CINCINNATI OBSTETRICAL SOCIETY.

CINCINNATI, March 15, 1895.

DR. C. A. L. REED presented two specimens and said: The first specimen to which I call your attention is that of a vermiform appendix. I was called to Hamilton a little over two weeks ago to operate for a strangulated hernia. I found the case to be that of a man of fifty-five, who had had a hernia for about ten years, a constant protrusion that had been irreducible, but that had given him no further trouble. Thirty-six hours before I was called the lump began to enlarge, the patient experienced a considerable amount of pain, had some persistent vomiting, but not of that extreme sort, however, which bespeaks obstruction. Upon examination I found the case to be one of direct inguinal hernia. The hernial sac was about the size of a hen's egg. I operated by making an incision obliquely across the sac, about two inches above Poupart's ligament, and as far down as was necessary to enable me to command all the conditions below. I then dissected out the sac, which I found to be adherent, and upon opening it there escaped about two ounces of fluid. The ring was very narrow and very tight. I found the contents of the sac were adherent, and I found it impossible to reduce the hernia, even after having divided the ring in the ordinary way. I therefore proceeded to do the operation which I have described heretofore, going into the pelvic cavity above Poupart's ligament, and commanding the hernia from above. Even by this means I found myself unable to reduce the hernia without exercising undue traction. But I was able to accomplish something I could not have accomplished from below without considerable difficulty; that was, I discovered that the contents of the sac was an enlarged and elongated vermiform appendix. I then dissected out the sac, inverted it and drew it out from above and removed the appendage, the apex of which you will find adherent to this specimen. After ligating the vermiform appendix at its base, I closed the sac over the stump and let the caput coli drop back. The case has made an uninterrupted recovery by first intention.

The other specimen was removed three days ago. It was a case of procidentia, the most complete I have ever seen. The protrusion extended more than six inches below the vulva. The cul-de-sac of Douglas was drawn down to in front of and below the fourchette. The bladder was external to the ostium vaginæ. A sound introduced into the urethra dropped directly downward and outward. In other words, the hernia of the pelvic organs was practically complete. I operated upon this case by a vaginal hysterectomy, and in doing so, I had recourse to a technique which I believe is applicable only in strictly non-malignant conditions of the uterus, namely, I left a circle of uterine tissue, by which means I was able to do the operation without either a clamp or ligature. There was no bleeding. After having done this, the operation being practically an external one, and being able to deal with it as I would with the stump of an amputation, I simply closed it completely by interrupted sutures, dressed it antiseptically, and reduced the protrusion. The only thing more about the case was the fact of the most profound shock I have had reported to me. She had not rallied when I left the hospital, but the shock seemed to be intensified and it was protracted during five hours, during which time her temperature dropped to nearly 95° in the axilla, and the pulse was practically imperceptible, although there was not an ounce of blood lost in the whole procedure. The doctor in attendance, however, administered some nitroglycerin and whisky hypodermically, when reaction came, and the patient regained her normal condition.

After the removal of the uterus, the opening into the peritoneal cavity seemed almost as pretentious as was the vagina itself. I did not remove the appendages in this case for two reasons: In the first place, they were healthy; and in the next place, I felt for the comfort of the patient, it would be well to defer the menopause until the physiological period, if that period could be hastened by the removal of the appendages. Then, too, the operation impressed me as affecting the patient considerably, there being profound shock. The interesting point is that you do secure very complete hemostasis by leaving this little segment of uterine tissue surrounding the wound.

DISCUSSION.

DR. R. B. HALL: I think there can be no question as to the advisability of total extirpation in a case presenting the condition of this woman. All other operations up to date have been failures, so far as permanent beneficial results for the patient are concerned. The old operation of high amputation of the cervix and plastic operations have not yielded satisfactory results. In other words, we have had a return of the former prolapsus. Whether or not total extirpation, with the support that can be given by bringing the pelvic fascia together, and the plastic operations to follow, will yield better results, remains to be seen. The operation is on probation. So far it has yielded better results and the patients have remained better than after the old operations. A patient in this condition is an invalid and a great sufferer, and demands relief.

I have made a number of operations for total prolapsus, as in this case, and so far none of the patients are further than four or five years beyond the operation, yet they all remain well. None are so far from the operation as to say they will not have prolapsus or hernia later, but the time is sufficient to demonstrate that it yields better results than the old plastic operations, and I am inclined to the opinion that it is going to be the recognized method of treatment in these cases.

As to the method of enucleation. Since I was convinced theoretically that this operation would stand the test, I have not had a suitable case upon which to practice it. In other words, I do not believe that it is advisable to make a vaginal hysterectomy without a clamp or ligature, by this method, in malignant disease. I think in cancer of the uterus as elsewhere we should keep as far away from the disease as possible, instead of keeping so close to it as we must in the method which has just been described. It is a feasible operation, but it must be confined to the cases with non-malignant disease, and that is a rare condition for vaginal hysterectomy. So, total hysterectomy without ligature or clamp is not going to be so universally adopted as the gentleman in Chicago would have us believe. The specimen is a very beautiful one and a very beautiful result has been obtained. Why the patient should suffer so from shock in this case I cannot understand from the description of the operation. I cannot think the shock was due to the method of operating, and if he had operated with a ligature or clamp it would have been just the same.

In vaginal hysterectomy, where you pull the uterus well down, some patients suffer more from shock than in any other operation with which I am acquainted. The method of closing the wound, I think, is the correct one. The tissues brought together in this way strengthens the pelvic floor; in this case it made practically a new pelvic floor; it shortens it by taking out the middle segments, and the patient is less apt to suffer prolapsus. I am satisfied after the plastic operation there will be no hernia through the pelvic diaphragm.

I wish to speak of one other point. In all cases of operations for prolapsus we have a relaxed condition of the pelvic floor necessarily from the preceding procidentia, and to make the pelvic floor shorter is a consideration. In all the operations I have made in this way, which are perhaps a half-dozen or more—I do not remember just the number—I removed the ovaries and tubes in every instance. I do not make any effort to dissect the tubes and ovaries out, but cut them off close to the uterus and then tie them, making a ligature as in abdominal incision. After removing the appendages, bring the stumps together, which makes them very tight—they will not always reach, but make them reach as far as they will—and then close the peritoneal cavity off with sutures. Bring the raw ends of the stumps into the vagina and the peritoneal edges of the stumps across, which blocks off the pelvic floor in a way.

DR. J. AMBROSE JOHNSTON: In regard to the case presented by Dr. Reed, I would say I wish to concur in his statements.

The cause of the prolapsus is probably to be found in the fact that the individual quite frequently walked up Sycamore hill, carrying heavy burdens.

On account of the extreme prolapse, it seemed that every organ in the pelvic cavity had lost its connection with the pelvis. The vagina was completely prolapsed, and the bladder accompanying it seemed to be wholly without the pelvis.

The operation went along smoothly, and for a while I thought the uterus would be completely extirpated without entering the peritoneal cavity.

Whether the operation will be a success or not is a question, because of the redundancy of vaginal tissue, and the very great laxness of the various uterine ligaments which, as I have said before, appeared to have lost all connection with the pelvis. However, it is to be hoped that the contemplated colporrhaphy and perineorraphy will be sufficiently supplemental to the primary operation to thus overcome this prolapse.

I have seen Dr. Reed operate on several other cases of complete prolapsus which did not have so much laxness of the vaginal and uterine ligamental tissues, and in which, after removing the uterus, the pelvic floor was better restored than in this case.

As to the shock, I had a case not long since in which there was absolutely nothing to do but saw through a very thick pedicle in a partial inversion of the uterus, through an os only sufficient to introduce two fingers, and my anesthetizer kept saying, "What on earth are you doing? This woman's pulse is almost imperceptible." I experimented to see, and I found whenever I got my finger up near the fundus of the uterus with the vulcellum and dragged down, I could produce the shock at pleasure. It seems to be due to pressure on the nerves. I have no doubt the doubling in of the folds has produced the shock in this case. I have had the pulse slow down and stay down until I took out the drainage tube. The reason the shock was prolonged in this case probably was because the operator included the nerves in the sutures, and the ligature would not strangulate them, but just irritated them. In this case it was a suture and not a ligature which constricted the nerves. I thought Dr. Hall would make my speech, but he did not quite come to it. If you take these prolapsed uteri out through the abdomen, and do an abdominal instead of a vaginal hysterectomy for these prolapsed uteri, I think the results will be probably better in the way of preventing future relapses. Dr. Reed states the tube was six or seven inches long. Now, imagine the slack which must be taken up when the uterus is taken out.

A vaginal hysterectomy I always did hate. I have never done it, and I don't think I ever will. We cannot remove the broad ligament as freely through the vagina as through the abdomen. In the vaginal operation we have to work through a little hole, and how are you going to do in a great big fleshy woman, with a long tube, like this? You must remove it, and make it shorten up by the production of scar tissue.

DR. REED: The most important point that has been raised in this connection is that of elevating and securing in its normal situation the pelvic diaphragm. It is the restoration of the pelvic diaphragm to its normal plane. Of course, that is what we are seeking to do. And we are seeking to do that by approximating, as far as possible, the separated margins, and we hope to do that by the cicatrical process in the site of enucleation. I have had not a little nor yet considerable experience. As compared with abdominal hysterectomy, it is child's play. This is practically an external operation. It bears some resemblance to that operation which the general surgeons speak of with such eclat and flourish of trumpets—the amputation of the extremity. I should hesitate very much to do an abdominal hysterectomy in this condition. While the condition of descent has been accomplished and has become established, the condition of ascent has not been accomplished; and all of us know who have attempted to replace these prolapsed uteri, it is extremely difficult to push them up to, to say nothing of above, their normal plane. I have had two experiences in the removal of uteri of normal size by the abdominal method, and it is my earnest petition to the Throne above that I shall not again be called on to repeat the experience. Notwithstanding the Trendellanberg position, and notwithstanding all the advantages which we secure from the later developments of technique, it is certainly one of the most difficult things I have attempted. In one case I succeeded, and in the other case, having secured the best degree of hemostasis possible, I bowed myself out with best grace possible.

DR. HALL: Do you believe it would have been an exceedingly difficult operation, then, to have made a vaginal hysterectomy in that particular case?

DR. REED: I don't know that I have anything to add. The point that has been raised is to my mind one worthy of consideration, but I believe that in all the cases I have had in my hands, I would very much prefer to effect the shortening of the broad ligaments by a secondary operation, rather than by the primary operation through the abdominal incision. I question the safety of the abdominal method. As a rule the primary statistics of vaginal hysterectomy, even in malignant disease, are better than the statistics of abdominal hysterectomy for even non-malignant disease. I think that rule will stand investigation. Of course, Dr. Johnston spoke with a great deal of truth, probably, when he said he had never had a death from the abdominal method, but if he will attempt the vaginal operation he will find it easier.

---

### THERAPEUTICS OF CHOLERA INFANTUM.

Three rules should be observed in the treatment of this disease. First, remove, if possible, the cause; second, treat symptoms which *per se* are liable to endanger the life of the patient; and third, sustain vitality. The first is to be fulfilled by restricting the diet and prohibiting food entirely at first. Not only will this prevent increase of the disease, but it will do much to diminish its severity. The ordinary drugs are of but little avail, especially if other measures are not also employed. The author is a strong believer in the value of rectal irrigations and stomach washings. For irrigation of the colon he uses plain cold water with a small amount of peroxide of hydrogen or hydrozone. The fluid is carried high up with a soft rubber catheter. He repeats the irrigation every two hours, if necessary, but has rarely failed to see improvement after the first few irrigations. He washes the stomach with the same solution, and seldom finds it necessary to repeat the operation, the vomiting ceasing, as a rule, after the first washing. He uses no antipyretics. If the temperature is high, and is not reduced by the cold irrigations, he bathes the body with alcohol.—DR. BLECH, in *N. Y. Med. Journal.*

